# Comprehensive Review on the Use of Biocides in Microbiologically Influenced Corrosion

**DOI:** 10.3390/microorganisms11092194

**Published:** 2023-08-30

**Authors:** Xin Shi, Ruiyong Zhang, Wolfgang Sand, Krishnamurthy Mathivanan, Yimeng Zhang, Nan Wang, Jizhou Duan, Baorong Hou

**Affiliations:** 1 CAS Key Laboratory of Marine Environmental Corrosion and Bio-Fouling, Institute of Oceanology, Chinese Academy of Sciences, Qingdao 266071, China; shixin@qdio.an.cn (X.S.); wolfgang.sand@qdio.ac.cn (W.S.); kritamathi@gmail.com (K.M.); zhangyimeng21314@163.com (Y.Z.); wangnan123@qdio.ac.cn (N.W.); duanjz@qdio.ac.cn (J.D.); brhou@qdio.ac.cn (B.H.); 2 Center for Ocean Mega-Science, Chinese Academy of Sciences, Qingdao 266071, China; 3 Open Studio for Marine Corrosion and Protection, Laoshan Laboratory, Qingdao 266237, China; 4 University of Chinese Academy of Sciences, Beijing 100049, China; 5 Institute of Marine Corrosion Protection, Guangxi Key Laboratory of Marine Environmental Science, Guangxi Academy of Sciences, Nanning 530007, China; 6 Aquatic Biotechnology, University of Duisburg-Essen, 45141 Essen, Germany; 7 Institute of Biosciences, University of Mining and Technology, 09599 Freiberg, Germany

**Keywords:** biocide, microbiologically influenced corrosion, bacteria

## Abstract

A microbiologically influenced corrosion (MIC) causes huge economic losses and serious environmental damage every year. The prevention and control measures for MIC mainly include physical, chemical, and biological methods. Among them, biocide application is the most cost-effective method. Although various biocides have their own advantages in preventing and treating MIC, most biocides have the problem of polluting the environment and increasing microorganism resistance. Therefore, it has stimulated the exploration of continuously developing new environmentally friendly and efficient biocides. In this review, the application advantages and research progress of various biocides used to prevent and control MIC are discussed. Also, this review provides a resource for the research and rational use of biocides regarding MIC mitigation and prevention.

## 1. Introduction

The biological activities of microorganisms and their metabolites can directly or indirectly participate in the electrochemical process of corrosion. This results in an accelerated corrosion process of metals, concrete and other materials. This activity is called microbiologically influenced corrosion (MIC) [1]. MIC was discovered first in 1910 [2], and it is now well known due to continuous improvements in understanding.

MIC widely exists in marine, oil fields, freshwater environments, soil and other natural environments, and is a major cause of metal failure and other materials [3,4]. MIC is not limited to a single corrosion mechanism, but also can interact with other corrosion processes, such as crevice corrosion and stress corrosion, resulting in the degradation of many materials, which in turn results in huge economic losses and serious environmental damage [5,6,7,8]. The direct economic loss caused by metal corrosion in the world is about 700–1000 billion dollars every year. The cost of corrosion is equivalent to 3–4% of each country’s gross domestic product (GDP) [9]. According to a 2013 survey report, the global corrosion cost is estimated to be $2.5 trillion, accounting for 3.4% of global GDP. Among them, the United States 2.7%, India 4.2%, Europe 3.8%, the Arab world 5.0%, China 4.2%, Russia 4.0%, Japan 1.0%, and the rest of the world accounted for 3.4% of the total global corrosion cost [10]. In 2014, the corrosion cost in China exceeded 2.1 trillion yuan, accounting for 3.34% of China’s GDP [11]. H.-C. Flemming states that MIC accounts for more than 20% of corrosion cost [12].

**Figure 1:**
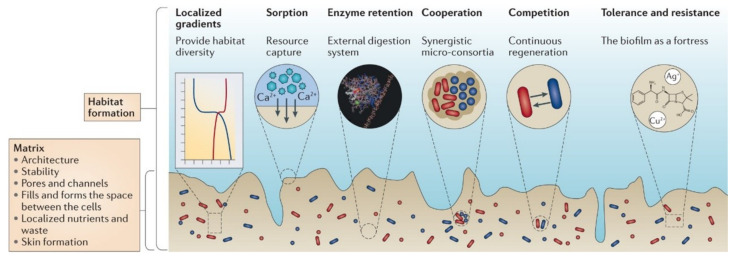
Emergent properties of biofilms and habitat formation [13]. Reproduced with permission from Springer Nature.

MIC is related to biofilm [4]. Microorganisms form a biofilm by secreting extracellular polymeric substances (EPS) composed of polysaccharides, proteins, nucleic acids, and lipids to attach and adhere to metal surfaces [13]. A biofilm can provide suitable conditions for the growth and metabolism of microorganisms. At the same time, it can also change the physical and chemical properties of the metal interface, thus affecting the corrosion process of a metal and the composition, structure, physical and chemical properties of the corrosion products (Figure 1). The corrosion effects of biofilms produced by different microorganisms on metal corrosion vary greatly; some may lead to an increase in the possibility and rate of metal corrosion [14,15]. Some studies have shown that biofilms can form a relatively dense protective film on the metal surface, thereby inhibiting metal corrosion, this phenomenon is also known as microbiologically influenced corrosion inhibition (MICI) [16,17]. The main mechanism of MICI may be due to the following reasons: (1) biofilms forms a diffusion barrier for corrosion products, inhibiting metal dissolution; (2) the aerobic microorganisms within the biofilms metabolize and consume oxygen, reducing the concentration of reactants on the material surface and slowing down corrosion; (3) compounds produced by microbial metabolic activities can inhibit material corrosion or inhibit the growth of corrosive microorganisms; and (4) some non-corrosive microorganisms can produce EPS to form a protective layer on the surface of the material [18]; The promotion or inhibition of material corrosion by biofilms is related mainly to the concentration, adsorption, charge, and three-dimensional structure of EPS components, even though some microorganisms may produce different EPS under different conditions and affect metal corrosion differently. Moreover, due to the many defense mechanisms of biofilms, the immobilized microorganisms in biofilms are usually more difficult to treat. Therefore, compared with planktonic microorganisms, the on-site treatment of immobilized microorganisms in biofilms requires 10 times or even higher doses of biocides [19].

**Figure 2:**
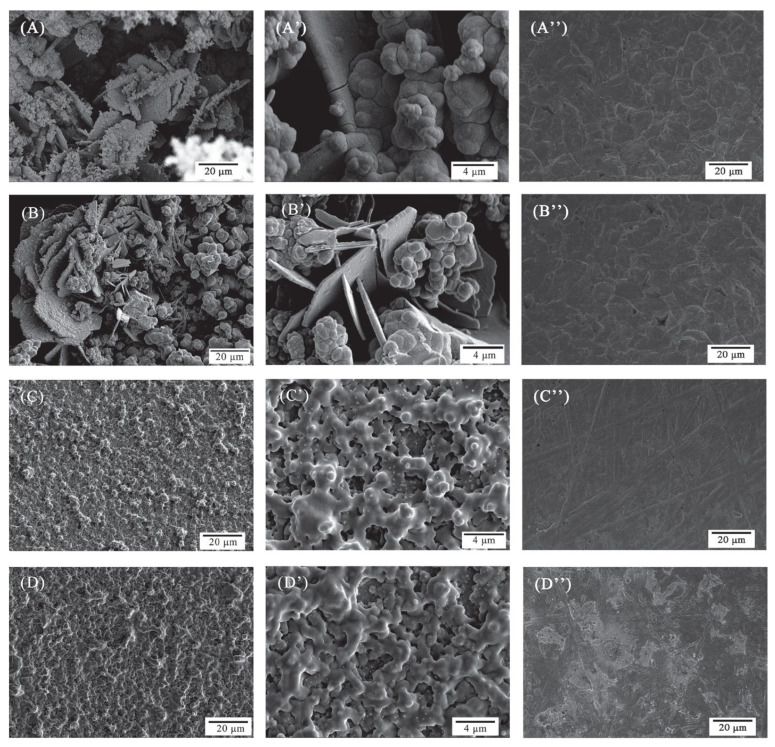
SEM images of Q235 of coupons after 8 days of immersion in abiotic (**A**–**A”**), *Desulfovibrio* sp. (**B**–**B”**), *Pseudoalteromonas* sp. (**C**–**C”**), and *Desulfovibrio* sp. + *Pseudoalteromonas* sp. containing (**D**–**D”**) media before (**A**,**A’**,**B**,**B’**,**C**,**C’**,**D**,**D’**) and after (**A”**–**D”**) the removal of biofilm and corrosion products [23]. Reproduced with permission from Elsevier.

Although biofilms are ubiquitous, studies have shown that the structure, composition, and spatial distribution of cells in biofilms of different devices in different locations, even in the same place, are different, and their corrosion behavior is also different [20,21]. This is a result of in the natural environment, where microorganisms adhere to the surface of materials mainly in the form of microbial communities, thus corrosion of materials by microorganisms is the result of the joint action of different microorganisms. Under the joint action of multiple microorganisms, they will exhibit different corrosion behaviors than single bacteria. Differences in material properties, nutrients, temperature, dissolved oxygen, and pH value can affect the abundance, diversity, and community structure of microorganisms, leading to different corrosion behaviors of microorganisms [22]. The mechanisms of mixed MIC mainly include synergistic and antagonistic effects. Synergistic action refers to, if two microorganisms coexist, one microorganism provides a suitable growth environment for the other microorganism through life activities, causing a large number of corrosive microorganisms to grow and accelerate the corrosion of materials. For example, most Sulfate-Reducing Bacteria (SRB) cannot survive under conditions of high oxygen concentration and, if coexisting with aerobic bacteria like *Pseudomonas* sp., the aerobic bacteria consume oxygen to creating an anaerobic environment for SRP and exacerbate metal corrosion (Figure 2) [23]. Antagonism refers to the inhibition of the growth of corrosive microorganisms due to competition between different microbial niches or the impact of other bacterial metabolic products of life activities, thereby slowing the corrosion of metals. The contribution of various microorganisms in biofilm to corrosion is relatively complex. In addition to the microorganisms that can promote corrosion and inhibit corrosion, some microorganisms may have no effect on corrosion, and they only use metal surfaces as support and utilize nutrients in the surrounding environment to grow [24].

Microorganisms related to corrosion include bacteria, archaea, fungi and algae. In actual production, MIC caused by bacteria and archaea mainly occurs in oil field systems, cooling water systems and marine environments. The types of bacteria related to metal corrosion include Sulfate-Reducing Prokar (SRP), Nitrate-Reducing Bacteria (NRB), Iron-Reducing Bacteria (IRB), Sulfur-Oxidizing Bacteria (SOB), Manganese-Oxidizing Bacteria (MOB), *Pseudomonas aeruginosa* (PA) and more [25,26]. Fungal corrosion is common in transportation systems and aerospace equipment; corrosive fungi mainly include molds and yeasts [27]. For example, the growth and propagation of molds in aircraft fuel systems will lead to the degradation of an aircraft’s fuel performance, fuel tank structure and other hazards, which can seriously affect flight safety [28]. Algae corrosion occurs mainly in freshwater environments with light, such as lakes and cooling water towers. The main algae causing corrosion are green algae, cyanobacteria and diatoms. In the freshwater environment, biofilm is mainly composed of algae under alternating day-night exposure conditions, which will cause corrosion of the material substrate [29]. The corrosion mechanism of different microorganisms on the same material may be different, and the corrosion mechanism of the same microorganism to different materials may also be different [30,31].

The prevention and control measures of MIC mainly include physical, chemical and biological methods. Among them, the use of biocides is the most cost-effective method [32,33]. At present, there are many kinds of biocides and their classification is confused. Therefore, the main characteristics, latest findings, existing problems and research progress of various biocides used for the prevention and treatment of MIC are reviewed in this paper. At the same time, this paper also provides a reference for the future research direction and rational use of biocides.

## 2. Treatment Methods of MIC Inhibition

### 2.1. Physical Methods

**Figure 3:**
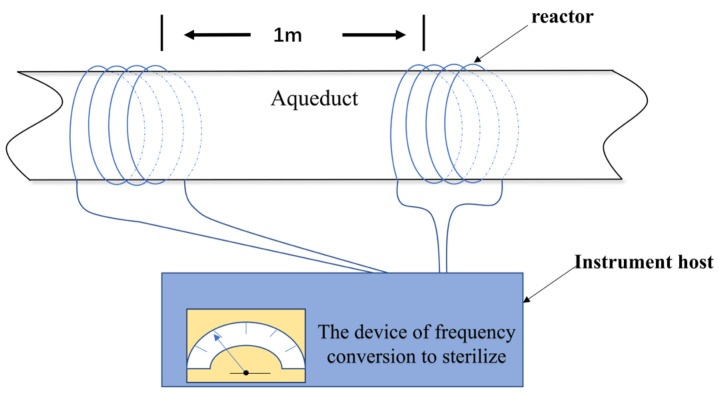
Schematic for the device of frequency conversion to sterilize.

Physical sterilization is widely used for its advantages of no pollution, no chemical residue, safety and environmental protection, and fast sterilization. Common physical sterilization technologies include ultraviolet sterilization, ultrasonic sterilization, frequency conversion sterilization, high-frequency current sterilization, etc. (Figure 3). Among them, ultraviolet sterilization technology is the most widely used in practical production [34,35]. In addition, mechanical cleaning can also be used to remove bacteria and biofilms and corrosion products on the surfaces of materials. However, due to the use of mechanical action, corrosive microorganisms can diffuse from the damaged biofilms spreading again. At the same time, the protective iron oxide deposition layer formed over a long period may be also removed, resulting in a matrix being re-exposed to the outside. Therefore, this technique may have exacerbated the corrosion [36]. Physical sterilization is often used in conjunction with chemical sterilization.

### 2.2. Biological Methods

**Figure 4:**
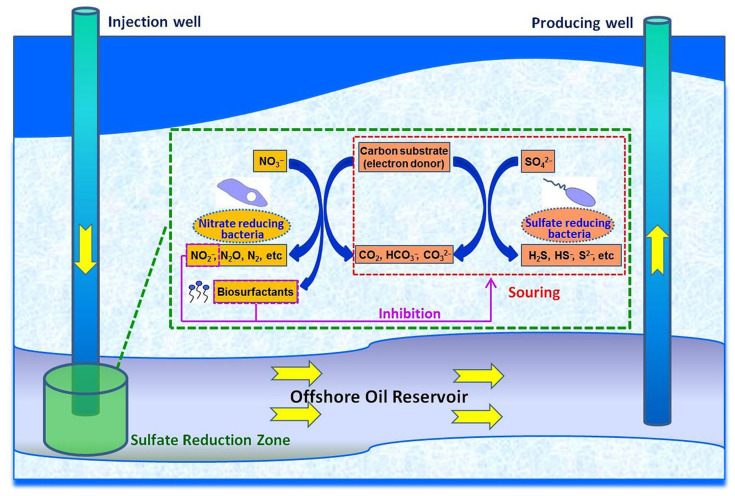
Schematic diagram of biosurfactant-involved NRB-SRB interactions in a souring reservoir system [37]. Reproduced with permission from Elsevier.

There are three main mechanisms for MIC prevention. One is to utilize specific bacteria that have the same niche as corrosive microorganisms, and do not cause corrosion. These have an advantage in competing with corrosive microorganisms for food and space, thereby inhibiting the activity of corrosive microorganisms. The second possibility means that some microorganisms can consume or convert SRP metabolites, such as $H_{2}S$, into other products that do not cause corrosion. Third, some microorganisms secrete substances that are harmful to corrosion causing microorganisms. These compounds may kill or inhibit their activity, reducing MIC. In some oilfields, nitrate is added to promote the growth of NRB to prevent MIC. Both NRB and SRP use acetic acid, lactic acid or long-chain fatty acid as energy sources. Since nitrate reduction produces more energy, ${\mathrm{NO}}_{3}^{-}$ has priority over ${\mathrm{SO}}_{4}^{2-}$ in the environment to be reduced [37]. So, NRB can inhibit the growth of SRP and reduce the generation of $H_{2}S$. The interaction between NRB and SRP in the oil layer is shown in Figure 4.

*Bdellovibrio* and Like Organisms (BALOs) and bacteriophages are a new type of corrosion prevention method, which have received attention in recent years. BALOs or bacteriophages can secrete various lysates to decompose cells, and even kill the sessile cells in the biofilm. In this way, the biofilm may be destroyed and MIC relieved [38,39]. Biological sterilization has the advantages of non-pollution, low cost and convenient post-treatment. At the same time, it can avoid the increase of bacterial resistance caused by the use of a large number of biocides. It is a promising alternative to conventional methods for inhibiting microbial corrosion. However, the use of microorganisms for sterilization is in its infancy; more research is needed for its application.

**Figure 5:**
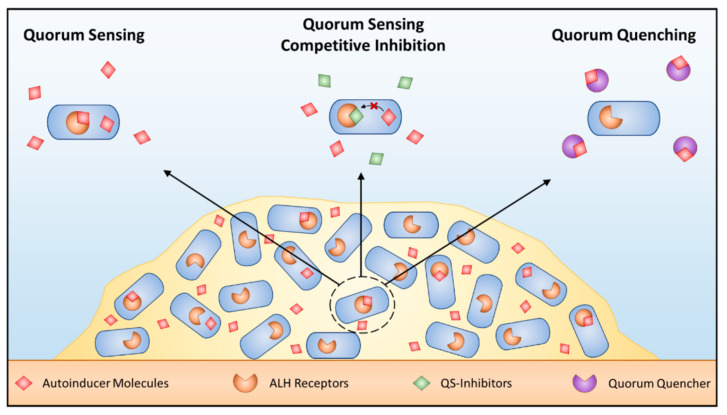
Schematic diagram of quorum sensing and quenching in a biofilm. AHL-dependent QS within biofilms (**left**) can be blocked using competitive QS inhibition that outcompetes AHL for AHL receptors (**middle**) or quorum-quenching enzymes that inactivate AHL signals (**right**). Reprinted with permission from Ref. [48].

Another direction is based on research concerning Quorum sensing (QS): the communication between bacteria that secrete signaling molecules and release them to extracellular cells to control their group behavior [40]. When the concentration of these signal molecules reaches a certain threshold, receptor proteins are activated, and the expression of corresponding genes starts, which regulates some corresponding behavior of microorganisms, such as bioluminescence [41], virulence factor production [42], biofilm formation [43] and plasmid transfer [44]. Microbes have a variety of signaling molecules. Common QS signal molecules include N-acylhomoserine lactones (AHLs), autoinducer-2 (AI-2), autoinducing peptides (AIPs), epinephrine/norepinephrine, etc. Different bacteria use different signal molecules for QS. AHLs are the dominant quorum-sensing signaling molecules in Gram-negative bacteria, while AIP are the dominant signaling molecule in Gram-positive bacteria [45]. The strategies for disrupting QS include receptor inactivation, enzymatic quorum quenching (QQ), and representative compounds such as QS inhibitors (QSIs) (Figure 5) [46,47,48].

Microorganisms mainly exist in the form of biofilms on various material surfaces [13]. QS is the core mechanism that plays a regulatory role in many processes within the biofilm and is closely related to the process of biofilm formation [49]. The biofilms make it difficult for microorganisms, such as bacteria, to come into direct contact with biocides, thereby reducing the efficacy of biocides. Some studies have shown that quorum sensing inhibitors can inhibit the formation of biofilms, making microorganisms more sensitive to biocides [50,51]. Therefore, QSIs can be used in combination as an enhancer of biocides to increase their effectiveness. It can also reduce the use of biocides and slow the development of microbial resistance [52,53]. QSIs have the advantages of high-biofilm-inhibitory activity, low toxicity, few drug-resistant microorganisms, and ecological friendliness [54]. At present, QS inhibitors mainly include furanones, flavonoids, sitagliptin and other types of compounds. The selection of QSIs should meet the following criteria: (a) a small molecule that can effectively reduce the expression of QS Regulator gene; (b) be highly specific; (c) be chemically stable, not easily degraded in the environment and by various host metabolic systems; (d) be more durable than natural AHL [55,56].

### 2.3. Chemical Methods

#### 2.3.1. Biocides

Biocides are a general term for chemical agents used to prevent and control the harm caused by various pathogenic microorganisms. Biocides contain substances that destroy cell enzymes. In this way, they kill corrosive microorganisms and destroy biofilms. They can be used to control MIC. In practical application, biocides are the most cost-effective method to prevent MIC [57,58]. However, a long-term use of a large number of chemical biocides will cause environmental pollution and increase the resistance of microorganisms to biocides. In addition, a long-term use of biocides will lead to changes in the microbial community in the pipe. The population structure of the new microbial community is unpredictable and may even increase corrosivity.

#### 2.3.2. Corrosion Inhibitors

A corrosion inhibitor can form an adsorption film on a metal surface, which may block the contact of microorganisms and their secretions with the metal. Thus, it is slowing down MIC. For example, in the past 20 years, the research groups of Monash University and Deakin University have studied some compounds of Rare Earth Metal (REM) and carboxylate as metal corrosion inhibitors. The corrosion inhibition mechanisms are that these compounds can form a thin protective films on the steel surface, increase the corrosion potential and significantly reduce the anodic kinetics [59]. However, many corrosion inhibitors have no obvious inhibitory effect on the growth of microorganisms on the surface of materials. some compounds can inhibit the growth of microorganisms. For example, tetracycline can block the secretion of EPS by Gram-negative bacteria to inhibit MIC [60]. Consequently, they cannot solve the problem of MIC. They often need to be used in combination with biocides [36,61].

#### 2.3.3. Overlay Protection

**Figure 6:**
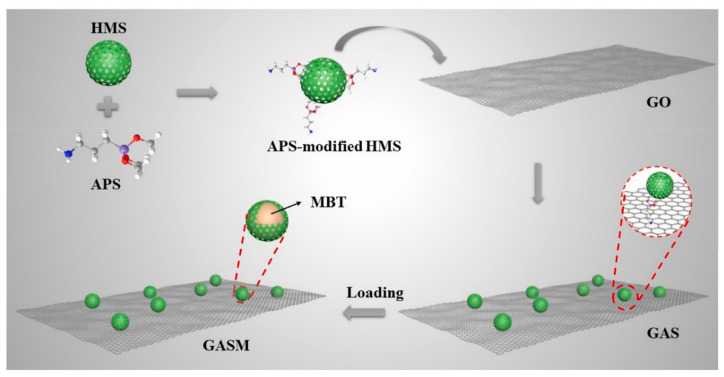
The synthesis process of coating GASM [62]. Reproduced with permission from Elsevier.

A coating can be used to separate the metal from corroding microorganisms. A coating can be used to smooth the metal surface to reduce the adhesion of microorganisms, thus preventing the formation of biofilms and, subsequently, MIC. GASM-Coat synthesized by Yan et al. can achieve self-healing in damaged areas and has good corrosion resistance in long-term impregnation [62]. A coating can also be designed to kill bacteria; for example, a coating obtained upon noncovalent immobilization of various side-chain degradable, water-insoluble quaternary polyethylenimine (QPEIs) polymers, obtained upon partial hydrolysis of QPEIs (thus containing both cationic and zwitterionic groups), can kill bacteria and inhibit the formation of biofilms (Figure 6). At present, most coatings are made from polyethylene and epoxy ides [62,63].

#### 2.3.4. Electrochemical Protection

Electrochemical technology is also commonly used to avoid metal corrosion or reduce corrosion, among which cathodic protection has a wide range of adaptability, adjustable protection current and potential and automatic monitoring and other advantages. In the marine environment, cathodic protection is usually used to prevent the corrosion of carbon steel by anaerobic microorganisms with remarkable effect. The principle is that by adding an external current or connecting with another more active metal, the protected metal is not easy to lose electrons and relieve MIC [64,65,66]. The results of Liu et al. show that cathodic protection does not affect the growth of planktonic cells, but has an effect on sessile bacteria on steel surfaces [67].

In terms of MIC prevention, researchers developed a variety of corrosion prevention methods, but biocides are the most direct, effective and widely used corrosion prevention in practical application. According to corrosion caused by microorganisms and the development direction of biocides in recent years, this paper summarizes the research progress of biocides used for the prevention and control of MIC. It aims at providing a reference for the research and rational use of biocides.

## 3. Biocides

Biocides often are used to mitigate microbiologically influenced corrosion (MIC) of construction materials in oil fields, delivery pipelines, water purification systems, etc. Commonly used biocides can be divided into oxidizing biocides and non-oxidizing biocides according to their mechanism of action.

### 3.1. Oxidizing Biocides

The most common oxidizing biocides are chlorine, bromine, ozone, and hydrogen peroxide. Oxidizing biocides are prone to react with mostly organic substances in the environment, resulting in an unstable bactericidal performance. In addition, the free radicals released by oxidizing biocides can increase metal corrosion, which is leading to equipment damage and environmental damage. Oxidizing biocides have no significant effect on inhibiting the formation of biofilms. The limitations of oxidizing biocides do not allow their widespread application in the field of industrial corrosion prevention. If using oxidizing biocides, the oxidizing ability, dosage, and treatment type of the biocide must be considered, and adverse effects should be evaluated carefully.

#### 3.1.1. Chlorine-Releasing Agents

Chlorine-releasing agents (CRAs) include chlorine, sodium hypochlorite and hypochlorous acid. Chlorine gas has abundant sources, low cost, high sterilization efficiency is easier to use, among other advantages. It was used widely in the past. Chlorine can release free radicals to attack cellular components and kill microorganisms [68]. However, due to its short protection time, poor stability and easy reaction with organic matter in the environment, it also pollutes the environment. Thus, its application is decreasing at present. In recent years, the research on oxidizing biocides has developed towards the direction of safe use and high sterilization efficiency.

${\mathrm{ClO}}_{2}$ has a stronger oxidation capacity than chlorine and usually does not produce toxic and harmful byproducts. Thus, it is a good alternative to chlorine disinfection. Shu [69] et al. showed that a moderate amount of ${\mathrm{ClO}}_{2}$ can effectively reduce the contents of bacteria and sulfide in oil field injection water, but excessive ${\mathrm{ClO}}_{2}$ will lead to increased metal corrosion. Therefore, it is necessary to strictly control the concentration of chlorine dioxide. ${\mathrm{ClO}}_{2}$ can react with the cell membrane, destroy the permeability of the cell membrane, and oxidize the -SH group of glucose oxidase to the -S-S group, destroying enzyme activity and bacterial death [70].

Compared with ${\mathrm{ClO}}_{2}$, NaClO promotes a slightly reduced degree of corrosion for material [71]. Therefore, in recent years, domestic oilfields have begun to replace ${\mathrm{ClO}}_{2}$ with NaClO. The mechanism of NaClO is that NaClO dissolves in water to form HClO and NaOH. HClO is a strong oxidant, and NaOH neutralizes with amino acids and plays a bacteriostatic role. NaClO can also oxidize EPS of biofilms, leading to EPS depolymerization, dissolution and separation [71].

#### 3.1.2. Bromine and Its Derivatives

The germicidal mechanism of bromine is similar to that of chlorine, but its germicidal effect, application conditions and environmental protection factors are better than chlorine. Thus, it is a good substitute for chlorine. Bromine-containing biocides are divided into oxidizing biocides and non-oxidizing biocides. Commonly used bromine-containing oxidizing biocides include bromine chloride, hypobromous acid and its salts, bromochlorohydrin, etc. [72]. Sodium bromide and sodium hypochlorite can be used at the same time, but the application is inconvenient and sodium hypochlorite is unstable. Therefore, sodium bromide is usually prepared as an aqueous solution and added to the water together with sodium hypochlorite. Alao stabilizers are added. In this way, sodium hypochlorite can oxidize bromine ions to obtain hypobromous acid and hypobromate ions, in order to achieve the goal of rapid sterilization [73].

#### 3.1.3. Ozone

Ozone has a wide range of sources, high bactericidal efficiency, broad spectrum, does not easily produce resistance, has reaction product safety and little environmental pollution. Ozone has strong oxidative effect. It can react with the cell membrane, cell walls, and EPS, etc., to destroy the structure, which results in increased permeability of the cell membrane, and the outflow of intracellular substances. This leads to cell dissolution and death. Secondly, it oxidizes biologically active compounds in the membrane (such as enzymes, lipoproteins, lipopolysaccharide, etc.), affecting the normal physiological function of cells [74]. Ozone dissolves in water, forming ozone water for sterilization. Microorganisms react directly with the dissolved ozone, and indirectly with the hydroxyl groups generated by the decomposition of ozone. Ozone has a high oxidative capacity and redox potential, therefore, it has broad-spectrum bactericidal properties. It is very effective against most microorganisms present in industrial systems and can kill microorganisms on the surface of biofilms. The residual concentration after sterilization is small and has anti-scaling performance [75]. However, the stability of ozone is poor; it is easy to decompose when used, efficacy time is short, and free radicals generated by ozone can attack the surface of materials, therefore, easily damaging the equipment.

#### 3.1.4. Hydrogen Peroxide

**Figure 7:**
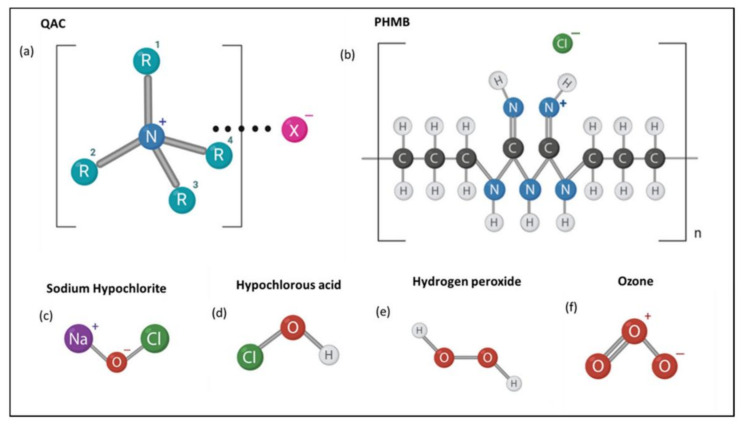
Molecular structures of common biocides. General structures for (**a**) QACs (Quaternary Ammonium Compounds); (**b**) polyhexamethylene biguanides (PHMB); (**c**) sodium hypochlorite; (**d**) hypochlorous acid; (**e**) hydrogen peroxide and; (**f**) ozone are depicted. Reprinted with permission from Ref. [76].

Hydrogen peroxide is a strong oxidizing biocide with high efficiency, high performance, convenient to use, pollution-free, non-toxic and minimally corrosive (Figure 7). Its bactericidal mechanism is similar to ${\mathrm{ClO}}_{2}$ and other strong oxidizing biocides. A proper amount of hydrogen peroxide can kill bacteria, spores, viruses and fungi. It is widely used in medical, military, industrial and other fields.

### 3.2. Non-Oxidizing Biocides

Various types of non-oxidizing biocides exist, including aldehydes, quaternary ammonium compounds, quaternary phosphonium salts, guanidine and heterocyclic compounds. These compounds have good bactericidal effects on microorganisms, good broad-spectrum properties, and good durability in the environment, making them more widely used in industrial fields. But like oxidizing biocides, many of these compounds are toxic and cause environmental pollution.

#### 3.2.1. Aldehydes

Aldehydes are non-ionic biocides. Their main mechanism of action is to penetrate cells, form complex precipitations with some components, such as proteins, inhibit bacterial metabolism, and achieve the killing of microorganisms or inhibiting their growth and reproduction [32]. Aldehydes are mainly organic aldehydes, such as formaldehyde, acrolein, glutaraldehyde, isobutyraldehyde, cinnamaldehyde, benzaldehyde, glyoxal, etc. Among them, glutaraldehyde is the most common.

**Figure 8:**
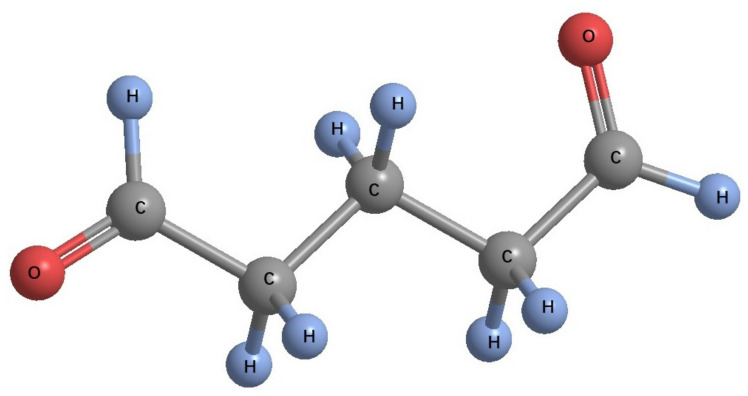
The structural formula of glutaraldehyde.

Glutaraldehyde (Figure 8) has advantages of high efficiency and rapid sterilization, a broad spectrum of sterilization and wide application environment. It has no corrosion effect on metal and other materials. However, glutaraldehyde is toxic. Thus, its application in practical production is limited. Glutaraldehyde’s bactericidal activity is mainly affected by concentration, temperature, pH and other factors, among which cationic surfactants have a synergistic effect on the bactericidal activity of glutaraldehyde. Combining it with decamethonium bromide or benzalonium bromide greatly improves its bactericidal activity [77].

#### 3.2.2. Quaternary Ammonium Compounds

**Figure 9:**
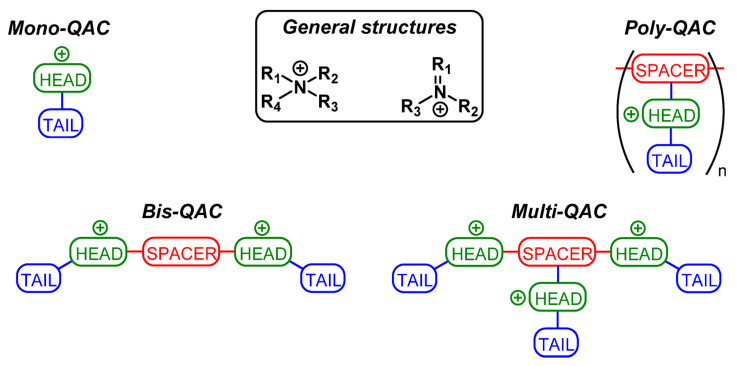
General structures and types of QACs. Reprinted with permission from Ref. [78].

Quaternary ammonium compounds (QACs) are very effective cationic biocides, which have been used widely in industrial cooling waters and oilfield water injection systems. QACs can be subdivided into Mono-QAC-QACs, Bis-QACs, Multi-QACs, and Polymeric Quaternary Ammonium Compounds (Poly-QACs) [78]. Their basic structure is shown in Figure 9. The structure of QACs consists of a positively charged nitrogen atom, three or four substituents, and a double bond. QACs use their positive charge to interact with the negative charge of the bacterial cell wall to form static electric bonds, allowing their penetration into the cell wall and a destructive interaction with the cytoplasmic membrane. This is followed by the leakage of intracellular components and, subsequently, cell death [79]. QACs can kill SRP growing under biofilms and destroy biofilms. In addition, QACs also have the effect of corrosion inhibition [80]. In order to obtain a quaternary ammonium compound with good biocide performance, the structure can be modified based on the original. At present, further research on these compounds is needed worldwide.

Mono-QACs have low bactericidal effects, but they have the advantages of low concentration, low toxicity and low cost, so still they are widely used [81]. Common Mono-QACs include dodecyl dimethyl benzyl ammonium chloride (DDBAC), dodecyl dimethyl benzyl ammonium bromide, dodecyl trimethyl ammonium chloride and tetradecyl dimethyl pyridine ammonium bromide. Lauryl dimethyl benzyl ammonium chloride is the most representative cationic quaternary ammonium salt used as a biocide. It is also known as 1227. Wang et al. [82] studied the antibacterial properties of DDBAC against three bacteria growing in an aircraft fuel system. The results showed that DDBAC has good antibacterial properties, with a minimum inhibitory concentration of 64 mg/L. In addition, the lone electron pairs of the nitrogen atoms in DDBAC can form coordination bonds with the empty orbitals of aluminum alloys, forming a tight chemisorption layer. This layer has a good corrosion inhibition effect.

Long-term use of Mono-QACs may cause bacteria to develop resistance and require the use of a more efficient biocide. In order to improve the antimicrobial activity of the Mono-QACs, Bis-QACs (or Gemini-QACs), which consist of two symmetric quaternary ammonium groups, were developed. Bis-QACs are composed of two hydrophilic groups, two hydrophobic groups and a connecting group. The connecting group in the molecular structure combines the two single QACs, thus the bis-QACs have good water solubility with improved bactericidal performance. They also have two positively charged $N^{+}$ atoms in the molecule. These increase the positive charge through induction, which is more conducive to the adsorption of biocide molecules on the surface of microbial cells. Thus, they change the permeability of the cell wall, leading to cell dissolution and death [81]. Consequently, Bis-QACs biocides have an extremely strong killing activity. They are widely used in oil fields and circulating water systems. 

Additionally, not only do they have a strong stripping effect, able to remove bacteria and biofilm growing on metal surfaces, but they also can block the channel between bacteria and metal surfaces through adsorption and film formation with the result of an excellent corrosion inhibition [83]. Migahed et al. [84] synthesized three Bis-QACs biocides and evaluated their performance as corrosion inhibitors for carbon steel in sulfide-containing oil well formation water. They found that their Bis-QACs biocides act as effective eco-friendly corrosion inhibitors. The inhibition efficiency increased when increasing the length of the alkyl group attached to the tertiary nitrogen atom. The germicidal efficacy of Bis-QACs biocides is hardly affected by temperature and pH. Zhu et al. [85] studied the antibacterial and corrosion inhibition performance of a Gemini surfactant with a semi-rigid spacer and chain length of 12, namely N,N′-(((1,4-phenylenebis(methylene))bis(oxy))bis(ethane-2,1-diyl))bis(N,N-dimethyldodecan-1-aminium) bromide (12-B-12). The results showed that 12-B-12 is a multifunctional surfactant with excellent antibacterial and corrosion inhibition performance even at 0.01 mM, and Gemini surfactant 12-B-12 has better corrosion inhibition ability than 1227. Zhou et al. [86] synthesized a novel quaternary ammonium biocide 4,4′-(1,5-pentylene dihydroxyl)bis(1-dodecylpyridine iodide) (PA-12). They studied the bactericidal properties of PA-12 against SRP and other corrosive microorganisms. The results showed that the minimum bactericidal concentration of PA-12 was one order of magnitude lower than that of the traditional oil field reinjection biocide 1227. The average minimum bactericidal concentration of PA-12 was 5.2 mg/L. PA-12 could maintain the stability of bactericidal activity against the changes in environmental temperature and pH.

Multi-QACs are compounds with three or more charged nitrogen atoms in a molecule, and poly-QACs are formed by the polymerization of monomeric QACs. The synthesis of these two types of biocides is complex and costly. There are currently little reports on them.

#### 3.2.3. Quaternary Phosphonium Salts

**Figure 10:**
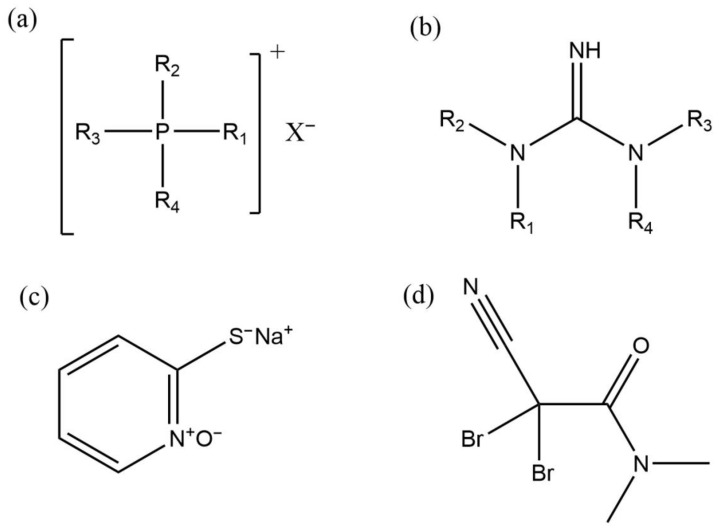
Molecular structures of common biocides. The general structures of (**a**) a quaternary phosphonium salt; (**b**) an alkyl guanidine; (**c**) SPT; (**d**) DBNPA.

Quaternary Phosphonium Salts (QPSs) are highly effective and broad-spectrum biocides (Figure 10). They were widely used in China in the early 1990s. The structure of a quaternary phosphonium salt biocide is similar to that of a QACs biocide. Also, these compounds have a substitution of nitrogen-containing cations like in QACs molecules by phosphorus-containing cations [87]. The bactericidal mechanism of a quaternary phosphonium salt is also similar to that of a quaternary ammonium salt. The cationic biocide with a positive charge is adsorbed on the negatively charged surface of bacteria, reacts with the cell membrane, changes its permeability, and destroys the activity of enzymes, which is causing the bacteria to die [88]. QPSs are relatively stable in chemical properties, do not react with general oxidation-reduction agents, acids and bases, have a low toxicity and a wide range of applicable pH. Also, they have a strong clay stripping ability, so they are very suitable for application in oil fields and other environments [89]. However, the N atom is the second-cycle element, and the P atom is the third-cycle element. The radius of the P atom is larger than the one of the N atoms, and the corresponding positive electric charge and the polarization it causes can easily adsorb on the cell membrane. Therefore, QPSs have a strong bactericidal ability [90].

The most commonly used QPSs biocide used in oilfield systems is Tetrakis(Hydroxymethyl)Phosphonium Sulfate (THPS) [91]. It is a water-soluble short-chain quaternary phosphonium compound that can be rapidly oxidized to trihydroxyphosphonium oxide (THPO), a compound of low toxicity to water. Therefore, THPS is a biodegradable and environmentally friendly oilfield biocide and can also be used as a sulfide remover. Xu [92] found that an insufficient bactericidal dose of THPS did not kill bacteria, but would stimulate the growth of bacteria in the biofilm, thus exacerbating corrosion. Liu et al. [93] prepared a polymeric triphenyl phosphonium salt and the corresponding low molecular-weight triphenyl phosphonium salt. Phosphonium trphenyl phosphonium salt has good germicidal performance against SRP and total general bacteria (TGB). However, low molecular-weight triphenyl phosphonium salt has a higher antimicrobial performance against SRP than polymeric triphenyl phosphonium salt. Liao et al. [94] prepared 1,12-dodecylidene bi (tributylphosphonium bromide) (DBTP) and applied them in the sterilization treatment of industrial water under the conditions of a 20 mg/L concentration and an action time of 0.5 h; the sterilization time by 1,12-dodecylidene bis (tributyl phosphonium bromide) against saprophytic bacteria was 98%, 96% for SRP, and 99% against iron-oxidizing bacteria.

#### 3.2.4. Guanidine Biocides

Alkyl guanidine is a cationic biocide. Guanidine (Figure 10), also known as iminourea (CH_5_N_3_), exists in natural products such as proteins, nucleic acids, streptomycin, and many kinds of plants such as beet, rice husk, mushrooms, and beans. There are also traces of guanidine in human and animal bodies. Free guanidine is very unstable, easy absorbs to carbon dioxide in the air and generates guanidine carbonate. Therefore, guanidine usually exists in the form of a guanidine salt [95]. Guanidine salts are an important component of guanidine compounds. After a hydrogen atom in guanidine is replaced or derived by an alkyl group, it becomes an alkyl guanidine surfactant with hydrophilic and lipophilic properties. These derivatives have good bactericidal properties. In recent years, with the in-depth study of these compounds, alkyl guanidine, as a non-toxic and efficient broad-spectrum biocide, has gradually attracted people’s attention.

The bactericidal mechanism of guanidine biocides is similar to that of QACs or QPSs biocides. The guanidine substances dissolved in water are adsorbed by negatively charged bacterial surfaces, destroying the integrity of bacterial cell membranes, denaturing the enzymes and other proteins in the bacterial body, which results in bacterial death [96]. The commonly used guanidine biocides are mainly long-chain alkyl guanidine, biguanides and polyguanidines.

There are many types of long-chain alkyl guanidine biocides, such as doxepin, polyhexamethylene biguanides (PHMB), carbendazim, etc. The traditional and economical method to prepare long-chain alkyl guanidine is to react monocyanamide with a corresponding fatty amine and add an acid (such as hydrochloric acid, acetic acid, etc.) to neutralize [97]. Biguanidine are organic compounds, which contain at least five nitrogen atoms and have a special bidentate structure. The biguanidine group has a variety of biological and chemical activities [98]. In order to obtain improved biocides, many scholars have begun to study polyguanidine biocides. Zhang et al. [99] synthesized a new type of polyguanidicide G-11 using guanidine hydrochloride and diethylene-triamine as raw materials. Their product showed that an excellent killing effect on SRP, total general bacteria (TGB), iron bacteria (FB) and various algae in industrial cooling water. The bactericidal effect is better than 1227 and bis-QACs or other conventional biocides. It has a long bactericidal action, strong killing power and a broad spectrum. Zhang [100] prepared the organoguanidine biocide SZ-58 and studied the effect of polymerization conditions on the properties of the product. The results showed that for a 50 mg/L dosage of SZ-58, the bactericidal action against SRP was 99%, and the slime stripping was 85%. With a dosage of 60 mg/L, SRP can be killed completely. In addition, together with SZ-58, the bactericidal effect and slime stripping effect are significantly better than with other conventional biocides. The on-site application was performed as a continuous dosing. SZ-58 has good compatibility with corrosion inhibitors and scale inhibitors in on-site water systems. For a dosage of 50 mg/L, the desulfurization effect was 100%, and the sterilization and desulfurization effects are obvious, which fully meets the on-site demand.

#### 3.2.5. Heterocyclic Biocides

Heterocyclic biocides refer to biocides that contain heteroatoms, such as N, O, and S, in the multicomponent ring of the molecular structure. There are many kinds of heterocyclic biocides, such as pyridine derivatives, imidazoline and polypyridine, thiazole derivatives, polyquinoline, isoxazole, and more on the market [101]. Common heterocyclic biocides are listed in Table 1 [102]. Heterocyclic biocides have the advantages of effective sterilization, environmental friendliness, wide adaptability to pH ranges, and good biocidal effects on bacteria, molds, algae, etc. Therefore, they are used widely in fields such as oil mining, paper mills, and cooling water systems. The mechanism of action of heterocyclic biocides is that after being absorbed by microorganisms, heterocyclic biocides will undergo catabolism in the mitochondria, which will significantly inhibit the synthesis of Adenosine triphosphate (ATP), thus destroying the energy supply of the microbial life system [102]; they also can utilize the active part of heterocycles to adsorb on the outer cell membrane of microorganisms, damaging their structure, affecting their normal life activities, and ultimately killing microorganisms [103].

A typical heterocyclic biocide is isothiazolinone. Isothiazolinone and its derivatives have biological characteristics such as bactericidal, antifungal and antiviral [104]. Therefore, it is widely used. With the unique heterocyclic structure and active part, isothiazolinone biocides effectively can destroy DNA molecules in bacterial cells and achieve the killing of microorganisms. In the late 1950s, studies on isothiazolinone compounds began. Since the 1960s, isothiazolinone biocides have been applied gradually in the industry [105]. The use of isothiazolone biocides alone or in combination with other biocides has an effective inhibitory effect on SRP under anaerobic conditions. Guiamet [106] showed that 50 and 100 ppm of isothiazolone biocide can kill 99% of sessile and planktonic SRP, but its killing effect on fungi in mixed biofilms is weak. Isothiazolidone is also effective against other bacteria such as *Pseudomonas* [107]. However, Bento [108] found that sub-effective doses of isothiazolone may instead lead to an increased growth of corrosion causing microorganisms. In acidic systems with high sulfide concentrations, isothiazolinones easily react with hydrogen sulfide. Thus, the biocide is no longer available for water injection containing hydrogen sulfide.

Sodium pyrithione (SPT) is a safe and environmentally friendly heterocyclic biocide (Figure 10). Its chemical structure is shown in Figure 9. SPT can damage the plasma membrane of microbial cells, which leads to membrane dysfunction and ion leakage. Carlson et al. [109] found that pyrithione can inhibit the growth of SRP and it is a potent inhibitor of sulfidogenesis in marine microbial community. SPT can react with iron ions to precipitates. These precipitates can form a protective film on the metal surface, which passivates the steel surface and achieves corrosion inhibition. Wang et al. [110] found that SPT has good antibacterial effects on both planktonic and sessile SRP. An 80 mg/L dose of SPT reduces concentrations of planktonic and sessile SRP on X80 carbon steel to undetectable levels.

#### 3.2.6. Organobromine Biocides

Organobromine biocides are a kind of industrial biocides that have arisen gradually in recent decades. The most representative of the non-oxidizing bromo-containing biocides is 2,2-dibromo-3-diazo-propanamide (DBNPA) (Figure 10). It is a new efficient organobromine biocide and water treatment agent with broad application prospects and development value. Compared with some existing disinfectants and biocides, DBNPA has the advantages of high efficiency, broad spectrum, easy degradation, low toxicity and more. At the same time, it has the multi-effect functions of sterilization, algae removal, viscosity reduction and corrosion inhibition [72]. The bromine-containing biocide has been used widely in water treatment, swimming pools, paper making, and other fields. DBNPA can undergo hydrolysis. The main products of DBNPA hydrolysis are dibromoacetic acid and dibromoacetonitrile. These hydrolysis products are more toxic or persistent than the parent compounds [111]. Therefore, this biocide has to be used with caution.

#### 3.2.7. Compound Biocide

Biofilm can relatively maintain the membrane’s internal environment stability and protect microorganisms from the toxicity of biocides. Therefore, sessile cells in biofilms are more difficult to eradicate than planktonic cells suspended in the liquid phase [112]. Killing sessile cells requires an increased concentration of biocide; it is difficult to achieve a good effect of killing microorganisms by using biocides alone [113]. Moreover, the actual production application environment is usually an open environment, where microorganisms are introduced constantly. Complete eradication of microorganisms all at once does not solve the problem and is not possible, and it would require a continuous dosing of biocides. Additionally, using the same biocide for treatment may promote resistance, which in turn leads to an increase in biocide dose over time, and excessive use of certain biocides may lead to scaling [114]. Therefore, different and efficient biocides are needed.

To improve the bactericidal performance of biocides, it becomes more and more difficult to achieve the purpose simply through developing new biocides. In addition, costs are increasing. Thus, there is a trend to improve the biocide performance by compounding; this method consists of mixing a variety of bactericidal substances to make them have a wide spectrum with efficient bactericidal properties. This is a common method, which is not only economical in practice, but also improves the effectiveness of killing microorganisms. Thus, the allocation ratio and method of compound production varies; their components need to be adjusted according to target environment and target microorganisms. Chang et al. [115] studied the biocidal and anticorrosive properties of new composite biocides thymol and benzyldimethyldodecylammonium chloride(BDMDAC). The results showed that the combination of phenolic with QACs exerted a strong antibacterial effect and can effectively slow the corrosion rate of metals. Composite biocides can also reduce the adhesion between cells and surfaces, reducing the formation of biofilms. Through synergistic metallic effects, the doses of thymol and BDMDAC to inhibit the growth of microorganisms in the combined treatment was significantly lower than using the substance alone.

In addition to mixing various biocides in proportion, some biocide enhancers can also be added to increase the efficiency. Biocide enhancers are chemicals that, if used alone, have no or only a weak killing effect on recalcitrant microorganisms. However, if used together with biocides, they can increase biocide efficacy and reduce biocide dosage [116]. Commonly used biocide enhancers include d-amino acids, norspermidine and chelators, etc. [14,117]. Wen et al. [118] used the chelator ethylenediaminedisuccinate(EDDS) and glutaraldehyde to treat biofilms containing SRP. The results showed that the combination of EDDS with glutaraldehyde significantly inhibits the formation of biofilms on metal surfaces. However, in their experiments, high concentrations of EDDS were used to demonstrate this concept. Therefore, it is necessary to use low-dose EDDS for testing to find the most suitable concentration of EDDS. Wen et al. [119] also tried to add 10–15% methanol to the dual combination of glutaraldehyde or THPS and EDDS to form a triple biocide cocktail. The research results showed that the composite biocide had a good killing effect on planktonic SRP. In addition, 50 ppm of glutaraldehyde, enhanced by 15% methanol and 1000 ppm EDDS, can remove an established biofilm.

In addition to the above-mentioned enhancers, non-biocidal Peptide A is a hot research topic in recent years. It has a cyclic 14-mer sequence (cys-ser-val-pro-tyr-asp-tyr-asn-trp-tyr-ser-asn-trp-cys) with its core being a 12-mer sequence. Peptide A cannot only inhibit the formation of biofilms at very low doses, but also destroy existing biofilms [116,120]. Antimicrobial peptides (AMPs) could form coatings by fixing on the surface of metal materials through a covalent bond, thus reducing the formation of biofilms. Based on a large number of known peptide sequences or by generating new peptide sequences, this method can target the AMP sequences of microorganisms to be killed, making it fast, simple, inexpensive, and environmentally friendly [121].

#### 3.2.8. Environmentally Friendly Biocides

**Figure 11:**
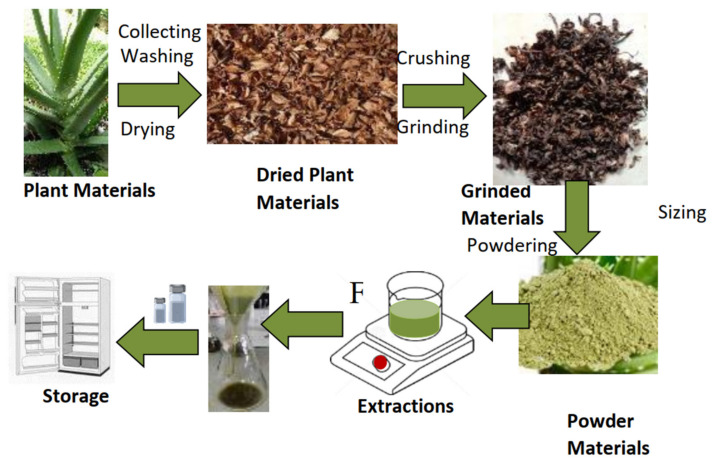
Diagram for plant extract preparing biocides. Reprinted with permission from Ref. [124].

Biocides are the most practical and effective means for preventing microbial influence on corrosion. However, since all biocides are chemical, these biocides are generally toxic; they can also react with substances in the environment and cause environmental pollution. Additionally, they increase the drug resistance of microorganisms. Therefore, an alternative eco-friendly biocide is needed to reduce such problems. In recent years, biocides from nature resources like plants have been developed. They are less-toxic, environmentally friendly, cost efficient, easily available, biodegradable and more [122]. They have great economic value in the field of future anti-corrosion engineering. The most extensive study on the inhibition and prevention of corrosion should be via exploring and developing green biocides from several plant extracts (Figure 11). The source of plant extracts for biocides screening is abundant [123]. Such phytochemicals can be used as biocides to inhibit the bacteria causing MIC. Some are phenolic compounds, limonoid compounds and flavonoid compounds [124].

**Figure 12:**
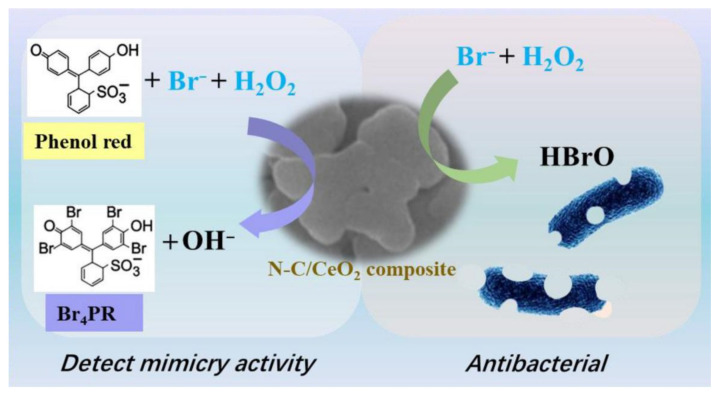
Bactericidal mechanism of N-C/CeO_2_ as haloperoxidase-like. Reprinted with permission from Ref. [130].

Hu et al. [125] synthesized Ag/Cu bimetallic nanoparticles (BNPs) with a particle size of 34 nm from ginger extract. The composite system had a dose-dependent bactericidal activity on SRP, and could reduce the generation of ferrous sulphide precipitation fouling. The SRP activity in culture can be reduced significantly by more than 50%, which means that low-concentration BNPs have dual functions of corrosion inhibition of carbon steel and SRP growth inhibition. *Polyalthia longifolia* extract is also an ecologically friendly biocide. Many compounds in the extract, such as furan and a lactone ring, have effective biological killing properties. They can destroy the cell membrane of SRP, cause cell death, and destroy the formation of biofilm [126]. Silva et al. [127] compared the performance of garlic oil and glutaraldehyde as biocides to prevent MIC. The results showed that both garlic oil and glutaraldehyde can reduce the corrosion of steel and prevent the formation of biofilm. Therefore, garlic oil is a promising natural biocide, which can replace commercial biocides with high toxicity. In addition to being used directly as a biocide to kill microorganisms and inhibit biofilm formation, some environmentally friendly compounds can also be used as biocide enhancers, such as trehalase, which is an enzyme that facilitates the transformation of trehalose into glucose through a biological catalyst, greatly enhancing the microbiocidal effect of THPS [128,129]. In order to simulate the process of marine algae self-secreting a haloperoxidase for sterilization, Wang et al. [130] synthesized N-C/CeO_2_ composite materials (Figure 12). The research results showed that the N-C/CeO_2_ composite as a biomimetic catalyst possesses haloperoxidase-like catalytic activity and can effectively catalyze the oxidative bromination of ${\mathrm{Br}}^{-}$ and $H_{2}O_{2}$ to generate HBrO. HBrO has strong oxidizing properties and can kill various corrosive microorganisms, effectively inhibiting MIC. This method indicates that artificial simulation of a natural product to synthesize biocides may be a new research direction for inhibiting biofilm growth and MIC.

In addition to extracting biocides from plants, microorganisms also can synthesize biocides. Microbially synthesized surfactants (biosurfactants) mainly fall into five categories: glycolipids, lipopeptides, phospholipids, polymeric compounds, and neutral lipids [131]. Biosurfactants have the advantages of low environmental pollution, low toxicity, high selectivity, and the ability to act in extreme environments [132]. Its mechanism of action is through foaming properties, which directly destroy the cell membrane and plasma membrane, causing cell lysis and death [133]. Therefore, it is difficult for microorganisms to develop resistance to biosurfactants [134]. For example, Bacillus subtilis can form biofilms on the surface of materials, and the EPS it produces contains biosurfactants. This compound can inhibit the growth of certain microorganisms such as bacteria, fungi, and viruses [135,136,137,138]. Therefore, biosurfactants may replace biocides and reduce the use of high concentration biocides and corrosion inhibitors in practical production applications, saving costs.

Nanomaterials are also environmentally friendly materials and, in recent years, they have gradually become substitutes for biocides due to their effective antibacterial effects [139]. Metal and metal oxide nanoparticles (NPs), carbon nanostructures, and other non-metallic fungicides are the main types of nanomaterials used as biocides [140,141]. NPs can attach to bacterial cell membranes through -NH_2_ groups, enhancing cell membrane permeability, leading to protein leakage and cell damage. Research has found that NPs can also affect the formation of biofilms [142,143]. Although nanomaterials are less toxic than biocides, long-term emissions of nanomaterials into the environment may pose a certain toxic risk to organisms [144]. Therefore, when using such biocides, the risk should be carefully evaluated.

## 4. Prospects

Biocides are the most commonly used and cost-effective method to prevent and control MIC. The application in actual production must make specific decisions according to the actual situation, and one must select biocides according to the target to prevent and control MIC. However, long-term use of the same biocide will cause environmental pollution and increase microbial resistance, thus reducing the efficiency of such biocides. They may lose their effect. Therefore, it is necessary to constantly develop new biocides. The future development aims at:

1. Multifunctionality. In addition to high efficacy and broad-spectrum bactericidal efficacy, biocides must have functions, such as corrosion inhibition and scale inhibition, to reduce the amount and input of various agents, avoid antagonism and reduce environmental pollution;
2. Mix of biocides. The development of new biocides requires huge economic costs. Thus, it is unreasonable to increase the dosage of drugs or develop new biocides frequently to solve the problem of microbial resistance. A reasonable combination of biocides can improve the sterilization efficiency and prolong the sterilization cycle. In addition, a variety of common chemicals, such as scale inhibitors and corrosion inhibitors, can be added to improve metal protection;
3. Increase use of physical and biological sterilization methods. The combined use of multiple technologies can enhance the advantages of various sterilization technologies, and also reduce sterilization costs, conserve energy and offer environmental protection;
4. Green and biodegradable compounds. The molecular design of new biocides needs to consider environmental toxicity and its impact on the ecosystem. Environmentally friendly biocides are required to reduce pollution.

## Figures and Tables

**Table 1:** Some common heterocyclic biocides. Reprinted with permission from Ref. [102].

Heterocyclic Type of Bactericide		Commonly Used Bactericides	Uses and Advantages
Ternary and quaternary heterocyclic compounds		Eopxiconazole, Temocillin,	Ternary heterocyclic compounds are often the key intermediates for the preparation of heterocyclic biocides. Temocillin is beta amide heterocyclic biocides with high fungicidal activity
Five-membered heterocyclic compounds	Thiazoles	Thiabendazole, Etridiazole Ethaboxam	Thiazole biocides possess a wide range of applications. In addition to inhibiting microbial corrosion, it can also be used to control harmful microorganisms in farmland
	Furan	Fuberidazole, Fenfuram Furalaxyl	Furan biocides have a wide range of biological activities, which can be used for sterilization, insecticidal and so on
	Azole	Pyrrolnitrin, Fenpiclonil Fludioxonil	Pyrrole biocides are derived from natural product nitropyrrolidins, among which imidazole methyl pyrrole compounds have the most prominent fungicidal activity
	Oxazoles	Ibotenic acid, Hymexazol Famoxadone, Glyodin Imazalil, Oxpoconazole	Oxazoles biocides have a good broad spectrum, bactericidal and permeability
	Pyrazoles	Pyrazophos, Furametpyr Pyraclostrobin	Pyrazole fungicide is a new type of heterocyclic biocides, which evolved from pyraclon
	Triazoles	Luotrimazole, Imazalil Bayleton, Diniconazole, Triadimenol	Triazole biocides have excellent bactericidal properties and a broad spectrum and are the most widely used biocides
Six-member heterocyclic compounds	Pyrimidines	Ferimzone, Mepanipyrim Cyprodinil, Pyrimethani	Pyrimidines are widely distributed and can be derived from a variety of natural extracts
	Pyridines	Fiuazinam, Sodium	The research and application of six membered cyclic pyridine
Benzimidazole		Benomyl, Carbendazim	Benzimidazole biocides have the advantages of broad spectrum and low toxicity

## Data Availability

No new data were created, or all data cited are available. Therefore, authorization is not required.
